# Antimicrobial Photodynamic Therapy against Endodontic *Enterococcus faecalis* and *Candida albicans* Mono and Mixed Biofilms in the Presence of Photosensitizers: A Comparative Study with Classical Endodontic Irrigants

**DOI:** 10.3389/fmicb.2017.00498

**Published:** 2017-03-30

**Authors:** Patrícia Diogo, Chantal Fernandes, Francisco Caramelo, Marta Mota, Isabel M. Miranda, M. A. F. Faustino, M. G. P. M. S. Neves, Marciana P. Uliana, Kleber T. de Oliveira, João M. Santos, Teresa Gonçalves

**Affiliations:** ^1^Faculty of Medicine, University of CoimbraCoimbra, Portugal; ^2^Department of Dentistry, Faculty of Medicine, University of CoimbraCoimbra, Portugal; ^3^Centre for Neuroscience and Cell Biology, University of CoimbraCoimbra, Portugal; ^4^Laboratory for Biostatistics and Medical Informatics, Faculty of Medicine, University of CoimbraCoimbra, Portugal; ^5^Department of Microbiology, Faculty of Medicine, University of PortoPorto, Portugal; ^6^Departamento de Química and Unidade de Investigação de Química Orgânica, Produtos Naturais e Agroalimentares, University of AveiroAveiro, Portugal; ^7^Department of Chemistry, Federal University of São CarlosSão Carlos, Brazil

**Keywords:** antimicrobial photodynamic therapy, endodontic biofilms, chlorin e_6_, *Enterococcus faecalis*, *Candida albicans*

## Abstract

Endodontic biofilms eradication from the infected root canal system remains as the primary focus in endodontic field. In this study, it was assessed the efficacy of antimicrobial Photodynamic Therapy (aPDT) with the Zn(II)chlorin e6 methyl ester (Zn(II)e_6_Me) activated by red light against monospecies and mixed biofilms of *Enterococcus faecalis* and *Candida albicans*. The results were compared with the ones obtained with Rose Bengal (RB), Toluidine Blue-O (TBO), the synthetic tetracationic porphyrin (TMPyP) as well as classical endodontic irrigants (3% NaOCl, 17% EDTA and 2% CHX). The antimicrobial efficacy of aPDT toward monospecies and mixed biofilms was quantified resorting to safranin red method. The changes of biofilm organization and of cellular ultrastructure were evaluated through several microscopy techniques (light, laser confocal and transmission electron microscopy). Zn(II)e_6_Me once activated with light for 60 or 90 s was able to remove around 60% of the biofilm’s biomass. It was more efficient than TBO and RB and showed similar efficiency to TMPyP and classical irrigants, CHX and EDTA. As desirable in a PS, Zn(II)e_6_Me in the dark showed smaller activity than TMPyP. Only NaOCl revealed higher efficiency, with 70–90% of the biofilm’s biomass removal. The organization of biofilms and the normal microbial cell ultrastructure were extensively damaged by the presence of Zn(II)e_6_Me. aPDT with Zn(II)e_6_Me showed to be an efficient antimicrobial strategy deserving further studies leading to a future clinical usage in endodontic disinfection.

## Introduction

Apical periodontitis is an inflammatory reaction of periradicular tissues caused by a microbial infection in the root canal system (Siqueira et al., 2000; Nair, 2006). Microbial biofilms are considered the major cause for primary and secondary root canal infection and the success of endodontic treatment relies on the effective eradication of such biofilms (Nair, 2006). Conventionally, this is accomplished by chemo-mechanical disruption with instruments and antimicrobial chemicals used topically inside root canals. However, current treatment strategies are insufficient to reduce microrganisms inside root canals below detection limits before permanent root filling. This is mandatory to achieve optimal healing conditions for the periapical tissues (Sjögren et al., 1997). Therefore, advanced disinfection approaches are required to effectively eradicate biofilms and increase the endodontic treatment success rate.

It is widely accepted that the main reason for endodontic treatment failure is the insufficient root canal microrganisms eradication (Siqueira et al., 2000). As residual species are not reachable to the host’s immune system, propagation and re-colonization is highly possible, endorsing microbial spread inside root canal system, which leads to endodontic infections. In 1965, apical periodontitis was recognized as a microbial mediated infection, which was later reinforced by ultrastructural microscopic techniques, revealing bacteria organized as biofilms within the infected root canals (Nair, 1987). Moreover, histopathological studies have also contributed to the concept that apical periodontitis is indeed a microbial biofilm-mediated disease (Carr et al., 2009; Ricucci et al., 2009; Ricucci and Siqueira, 2010).

As described by Donlan and Costerton (2002), a biofilm is a microbially derived sessile community characterized by cells that are irreversibly attached to a substratum or interface or to each other, are imbedded in a matrix of extracellular polymeric substances that they have produced, and exhibit an altered phenotype with respect to growth rate and gene transfer. In addition, it is accepted that microbial cells comprising the biofilm are more resistant than the planktonic counterparts (Donlan and Costerton, 2002) and multispecies or mixed biofilms are more resistant to drugs than monomicrobial biofilms (Costerton et al., 1999). As such, polymicrobial biofilms diseases are associated with worse clinical outcomes than monomicrobial infections for decades (McKenzie, 2008). Although the endodontic biofilm is constituted by multiple microrganisms (Tan et al., 2015), most of *in vitro* studies have been made in monospecies biofilms of bacteria or combined with *C. albicans* (Sabino et al., 2015).

The problem of endodontic biofilms eradication from the infected root canal system remains as the primary focus in endodontic field. In recent years, photodynamic therapy (PDT) has been applied with success in several types of cancers (Ochsner, 1997; Gomes et al., 2015), age-related macular degeneration (Kawczyk-Krupka et al., 2015) and also in the photoinactivation of several microrganisms (Almeida et al., 2014), called antimicrobial photodynamic therapy (aPDT). In the endodontic field, aPDT has emerged as an optional extra to classical irrigation solutions in root canal asepsis (Bonsor et al., 2006a,b) such as sodium hypochlorite (NaOCl), chlorhexidine gluconate (CHX) and ethylenediamine tetraacetic acid (EDTA). The NaOCl solution is the most widely used in endodontic treatment (Siqueira et al., 2007; Mohammadi, 2008; Vaziri et al., 2012; Wang et al., 2015) albeit with some degree of toxicity. To avoid this toxicity, other root canal asepsis approaches with lower or insignificant toxicity should be implemented. Therefore, aPDT has emerged with promising experimental results, anticipating a possible new era in endodontic disinfection (Siddiqui et al., 2013; Chrepa et al., 2014).

Photodynamic therapy involves the combination of a non-toxic photosensitizer (PS) with a harmless visible light source in the presence of oxygen. After being excited by light, the PS releases its energy or electrons to molecular oxygen producing highly reactive oxygen species (ROS) such as singlet oxygen (^1^O_2_), which induce microrganism’s injury and death, ideally with no host cell damage. Also, it has been indicated as bearing a strong potential in the fight against antimicrobial resistance (Hamblin and Hasan, 2004; Tavares et al., 2010; Costa et al., 2011). aPDT has also been studied as an auspicious approach to eradicate oral pathogenic microrganisms that cause, not only endodontic diseases, but also periodontitis, peri-implantitis, caries lesions and mucositis (Diogo et al., 2015).

**FIGURE 1:**
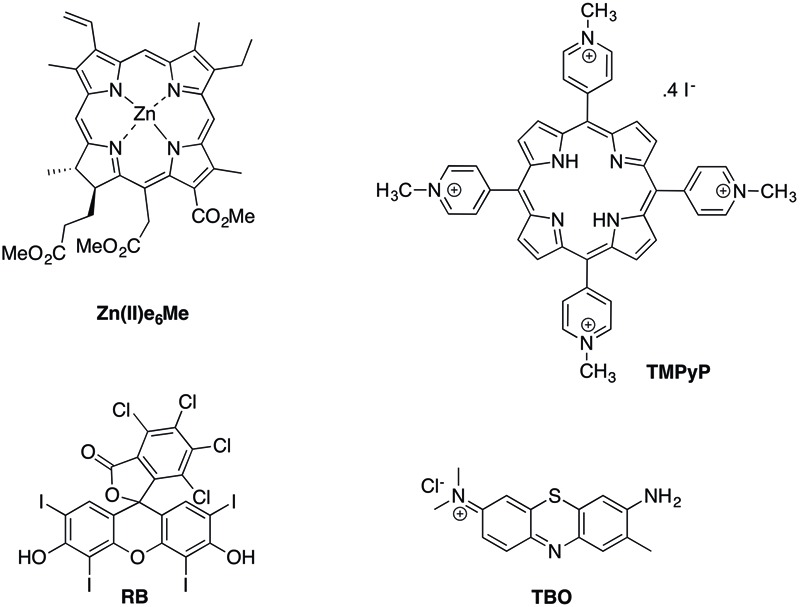
**Chemical structures of the photosensitizers tested**.

In this study, we analyzed the aPDT efficacy against monospecies and mixed biofilms of *E. faecalis* and *C. albicans* using the following PSs: toluidine blue (TBO), rose bengal (RB), a synthetic porphyrin 5,10,15,20-tetrakis(1-methylpyridinium-4-yl)porphyrin (TMPyP) and Zn(II)chlorin e_6_ methyl ester (Zn(II)e_6_Me) obtained from chlorophyll a (**Figure 1**). The antimicrobial results obtained by aPDT approach were compared with the ones achieved with three endodontic classical irrigants 3% NaOCl, 2% CHX, and 17% EDTA toward *in vitro* biofilms.

## Materials and Methods

### Strains and Media

The strain of *C. albicans* (YP0037) used in this study was obtained from the Pathogenic Yeast Collection of FMUC, University of Coimbra. *E. faecalis* (ATCC29212) was purchased from the American Type Culture Collection (ATCC). Microrganisms were stored at -80°C in 25% glycerol. When needed, pre-cultures were prepared by defrozen microbial cells in appropriate media, brain-heart infusion (BHI) for *E. faecalis* and YPD (0.5% yeast extract, 1% bacto-peptone, and 2% glucose) for *C. albicans*. For *C. albicans* growth it was used YPD broth. *E. faecalis* growth and biofilm formation was obtained in BHI liquid medium (Difco, Detroit, MI, USA). *C. albicans* biofilms and mixed biofilms consisting of *C. albicans* with *E. faecalis* were obtained in RPMI 1640 (Roswell Park Memorial Institute) medium (R8755, Sigma–Aldrich^®^).

### *In vitro* Biofilm Formation

For *E. faecalis in vitro* biofilm formation, bacterial cells were previously grown in 4 mL of BHI overnight, at 37°C. These cells were harvested by centrifugation (Biofuge Fresco, Heraeus, UK), at 16,000 *g* during 5 min at 4°C, and washed twice in sterile BHI. A bacterial cell suspension with a density of 1.5 × 10^8^ cells/mL (0.5 McF of McFarland scale) was obtained; 200 μL of this suspension was pipetted to each well of sterile 96-well polystyrene microtiter plates (Nunc F, Nalgene, Denmark). These plates were covered and sealed with parafilm, and incubated during 48 h at 37°C without agitation.

For the preparation of *C. albicans* biofilm, a loopful of cells from the solid stock cultures was used to inoculate 20 mL of YPD and incubated overnight in an orbital shaker (120 rpm) at 30°C. The cells were harvested by centrifugation (16,000 *g* for 5 min at 4°C), and washed twice with phosphate buffered saline (PBS). The final pellet was resuspended in pre-warmed RPMI-1640 at 37°C. The resulting cell suspension was diluted in RPMI to obtain a final suspension with a cell density of 1.0 × 10^6^ cells/mL. This was used to prepare *C. albicans* biofilms in 96-well polystyrene microtiter plate (Nunc F, Nalgene, Denmark). For that 200 μL of *C. albicans* suspension was pipetted to the plate wells. After sealing with parafilm, they were left to incubate during 48 h at 37°C, without agitation.

For the mixed biofilm of *E. faecalis* and *C. albicans*, the two microbial species were pre-grown overnight and prepared as described for the monospecies biofilms except that they were resuspended in 37°C pre-warmed RPMI-1640 (R8755, Sigma–Aldrich^®^). The two microbe suspensions at a concentration of 1.0 × 10^6^ cells/mL were mixed in pre-warmed RPMI-1640 in 1:1 ratio and incubated at 37°C, during 48 h to allow biofilm formation.

### Biofilms Eradication with Classical Irrigants

The classical irrigants tested were 3% NaOCl, 17% EDTA, and 2% CHX (CanalPro^TM^- endodontic irrigating solutions, Coltene). The biofilms were exposed to the irrigants for 60 and 90 s. A longer period of irrigation (30 min) was also tested because some authors defend that a continuous irrigation and time are important factors for the efficacy of classical irrigating solutions (Bystrom and Sundqvist, 1985; Haapasalo et al., 2010). After each period, the supernatants were removed and the chemical reactions were stopped using adequate inhibitors: 200 μL of sodium thiosulfate (S7026, Sigma–Aldrich) was added to the NaOCl treated group; 3% Tween 80 (T2575, Sigma–Aldrich) was used to neutralize CHX. Finally, 200 μL sterile distilled water was applied to dilute the 17% EDTA. Controls were made without the irrigants, in which the stop solutions were added, proving that these stop solutions, especially 3% Tween 80, did not interfered with the biofilm biomass quantification.

### Photodynamic Inactivation of Biofilms

In the aPDT experiments all the PSs tested (TBO, RB, TMPyP, and Zn(II)e_6_Me) were used at the same concentration (0.1 mg/mL). This concentration was chosen based on market formulation FotoSan agent^®^, in which the active substance is TBO at 0.1 mg/mL. This formulation is available in the dentistry market with a light source device (FotoSan^®^: 630, CMS Dental A/S, Glyngore, Roslev, Denmark) (Rios et al., 2011). The cationic porphyrin TMPyP and the modified chlorophyll, Zn(II)e_6_Me, were synthetized and isolated according to the literature (Carvalho et al., 2010; Menezes et al., 2014). Their ^1^H NMR and UV–vis spectra were consistent with literature data and their purity was confirmed by thin layer chromatography and ^1^H NMR (data not shown). TBO and RB used were purchased from Sigma Aldrich (T3260 and 330000-1G, respectively). Stock solutions (10 mg/mL) of each porphyrinic derivative (TMPyP and Zn(II)e_6_Me) were prepared in dimethyl sulfoxide (DMSO). For biological assays, the stock solutions of photosensitizers were diluted to the final concentrations in PBS.

**FIGURE 2:**
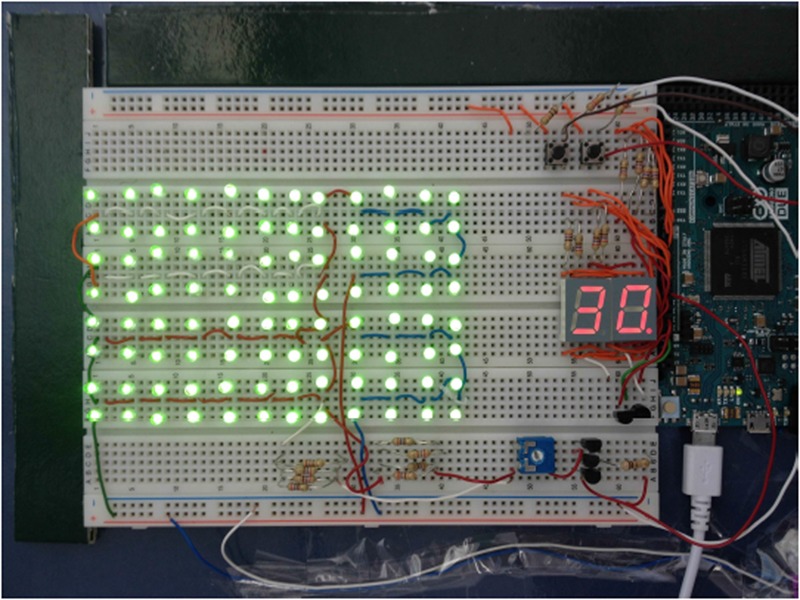
**Model of the experimental light emitting diode (LED) sources [green light diode (GaP); 557 nm of wavelength]**.

The irradiations of the PSs in the aPDT experiments were performed in the presence of adequate light emitting diode (LED) source setup to comply with the 96-well plates used in this study (**Figure 2**). The LED sources were built at request by the Telecommunications Institute – Informatics, Electronics and Telecommunications Engineering Department of the University of Aveiro, Portugal. RB was irradiated with a green LED with a wavelength peak centered at 557 nm, made with gallium phosphide pure (GaP), an output of 62.5 mW, continuous waves, density power of 42 mW.cm^-2^, energy fluence of 3780 J.cm^-2^, voltage of 2.5 V. TBO, TMPyP, and Zn(II)e_6_Me were irradiated with a red LED device with a wavelength peak centered at 627 nm, a gallium arsenide phosphide on gallium phosphide (GaAsP/GaP), with an output power of 75 mW, continuous waves, density power of 35 mW.cm^-2^, energy fluence of 3150 J.cm^-2^ and a voltage of 2.5 V.

The experimental methodology included a pre-incubation period of the biofilms with the PSs, in total absence of light, for 15 min, to allow the entrance of PSs into the cells (Diogo et al., 2015). After that, light activation was performed for 60 or 90 s. Also, in each assay it were included controls in which the PSs were not added, to study the impact of irradiation in the biofilms.

### Biofilm Biomass Quantification

The biofilm biomass was quantified using the safranin red (SR) assay (Kueng et al., 1989). After each experiment, 200 μL of methanol was added to each well of the 96-well plate. After 15 min, the content of each well was aspirated and let to dry. After drying, 0.1% SR solution was added and incubated for 20 min. The resulting solution was removed with a Pasteur pipette and two washes were made with distilled water. Two hundred microliter of acetic acid 33% (v/v) was added and 20 min after the absorbance was measured at 590 nm on a microplate reader (SpectraMAX Gemini XM, Molecular Devices, USA). The results were expressed as a percentage of biofilm removal when compared with the biomass quantified before irradiation or before exposure to the irrigants.

### Microscopic Study of the Biofilms

*E. faecalis* and *C. albicans* were grown and prepared as described above. One mL of the final suspensions was added to sterile 12-well polystyrene microtiter plates with glass coverslips (CBAD00120RA1#1.5, ThermoScientific-Menzel) coated with poly-D-lysine (Sigma-Aldrich^®^, P1149). After seeding, the entire microtiter plate was covered and sealed with Parafilm^®^and incubated for 48 h at 37°C without agitation. For confocal fluorescence microscopy, fresh cultures of biofilms were used. *E. faecalis* was stained with Syto 13 Green Fluorescent Nucleic Acid Stain (ThermoFisher Scientific^®^). *C. albicans* in monospecies biofilm was probed with polyclonal primary antibody Acris Antibodies Gmbh^®^RGTX40096 with anti-rabbit secondary antibody Alexa Fluor^®^594 (Invitrogen^®^, RA21207). Images were obtained with a Carl Zeiss Cell Observer Spinning Disk with Alpha Plan-Apochromat objective, at a magnification of 100×. For light microscopy, it was used an Olympus BX-40 microscope at 400× total magnification. Images were recorded on an Olympus C-200 digital camera.

For transmission electronic microscopy (TEM), samples of 48 h-biofilms were fixed with 2.5% glutaraldehyde in 0.5 M sodium cacodylate buffer (pH 7.2) for 2 h. Post-fixation was performed using 1% osmium tetroxide for 1 h. The samples were then rinsed with the same buffer, and dehydrated in a graded ethanol series (30 to 100%). Then, they were impregnated and embedded in Epoxy resin (Fluka Analytical). Ultrathin sections (∼70 nm) were mounted on copper grids (300 mesh) and stained with uranyl acetate 2% (15 min) and 0.2% lead citrate (10 min). Observations were carried out on a FEI-Tecnai G2 Spirit Bio Twin transmission electron microscope at 100 kV.

### Statistical Analysis

Data were analyzed using Prism (version 5) software (GraphPad Software, Inc., La Jolla, CA, USA). Statistical differences between groups were assessed with the independent samples student’s *t*-test or Mann–Whitney test and a significance level of 0.05 was assumed.

## Results

### Biofilm Removal

Before initiating the comparative study of the efficacy of aPDT and classical irrigants in the clearance of biofilms, it was important to verify if the PSs selected, TBO, RB, TMPyP, and Zn(II)e_6_Me, had the ability to disturb the biofilms in the dark (i.e., in the absence of light activation) at the same concentration used in the aPDT studies. It was clearly desirable that the PSs had zero or very low activity in total absence of light indicating that aPDT efficacy resulted strictly from the ROS generated by PS light activation. The results obtained from the biofilm biomass analysis, using the SR assay, showed that upon 15 min of exposing the biofilms to the different PSs, in the dark, there was a decrease of the biofilms biomass in values ranging from 5.7 to 16.6% (**Table 1**). Following a pre-incubation period in the dark with the PSs, each preparation was irradiated with the appropriate LED light. Thus, TBO, TMPyP, and Zn(II)e_6_Me were irradiated with a wavelength of 627 nm while 557 nm was used for RB. Three periods of irradiation were tested, 60, 90 s and 30 min. Since there were no differences between the 90 s and the 30 min periods, this longer period was abandoned (data not shown). Also, the controls of the impact of light irradiation in the biofilms during the 60 or 90 s of irradiation showed no damage of the biofilm, as assessed by the SR assay and by microscopic observation of the biofilm morphology (results not shown).

**Table 1:** Photosensitizers (PSs) effect in biofilm biomass^(1)^ in the total absence of light during an incubation period of 15 min.

Biofilm	TBO (%)	TMPyP (%)	Zn(II)e_6_Me (%)	RB (%)
*E. faecalis*	15.2	13.1	12.7	14.7
*C. albicans*	16.6	15.5	8.6	10.0
Mixed	12.4	10.0	5.7	7.5

*^(1)^The quantification of biofilm biomass was obtained with the SR assay and results are expressed as a percentage of biofilm removal when compared to the control biofilm biomass (*n* = 3).*

**FIGURE 3:**
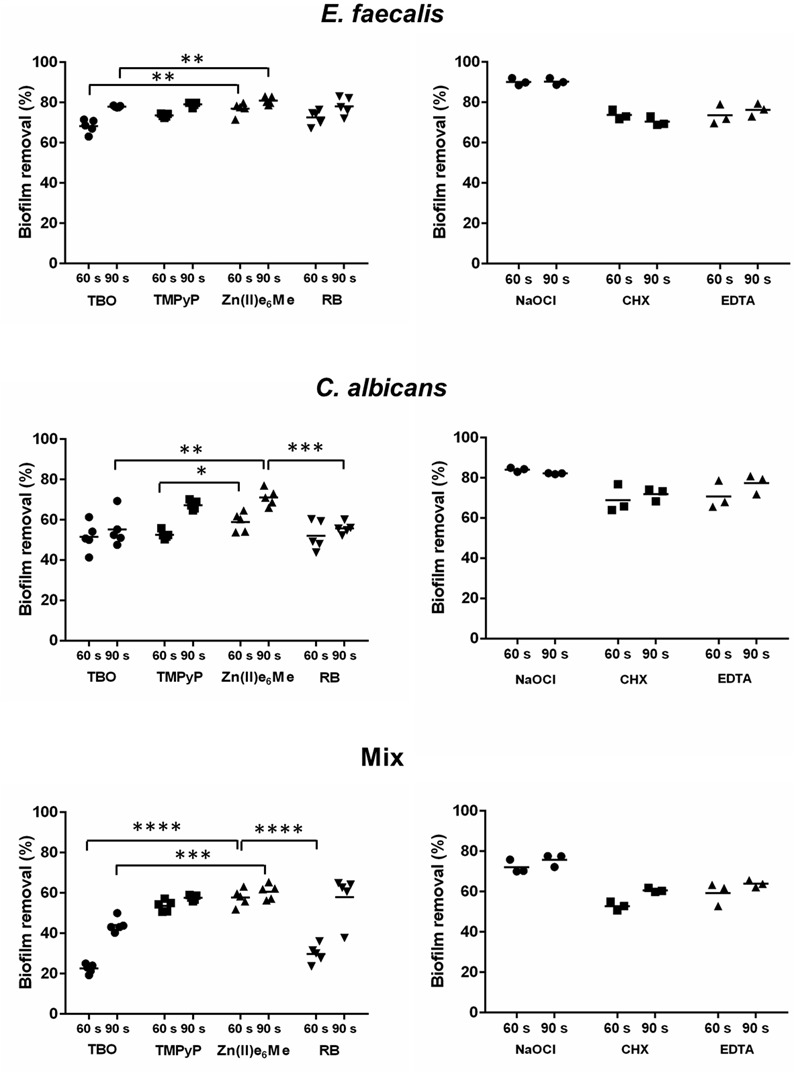
**Biofilm biomass removal upon antimicrobial photodynamic therapy (PDT) and treatment with classical irrigants.** Monospecies biofilms and mixed biofilms of *E. faecalis* and of *C. albicans* were treated by a PDT using several photosensitizers (left panels) and classical irrigants (right panels). The quantification of biofilm biomass was performed with the safranin red (SR) assay. The percentage of biofilm biomass loss was calculated in relation to untreated biofilm. Differences were analyzed by Student’s *t*-test using Prism software and considered significant at *P*-values of < 0.05. ^∗^*P* < 0.05; ^∗∗^*P* < 0.01; ^∗∗∗^*P* < 0.001; ^∗∗∗∗^*P* < 0.0001.

The results summarized in **Figure 3** (upper left panel) showed that Zn(II)e_6_Me, is more effective in the removal of *E. faecalis* biofilm than the other PSs used, in both irradiation periods (60 and 90 s) (*P* = 0.0079). Similar reduction values of *E. faecalis* biofilm were obtained using TMPyP and RB (**Figure 3**; upper left panel). For *C. albicans* biofilm, Zn(II)e_6_Me and TMPyP had similar efficacies in decreasing biofilm biomass upon 90 s of irradiation. However, a shorter period of irradiation, 60 s, revealed significant differences between the efficacies of both dyes in the capacity to remove biofilm biomass (**Figure 3**; middle left panel). Otherwise, Zn(II)e_6_Me was much more effective than the other PSs used, TBO (*P* = 0.0317), and RB (*P* = 0.0079) after 90 s of irradiation and at the same concentration.

The mixed biofilm seems to be less susceptible to aPDT than the monospecies biofilm, especially when using TBO as PS, (*P* = 0.0013), (**Figure 3**; lower panel). In this mixed community of *E. faecalis* and *C. albicans*, Zn(II)e_6_Me demonstrated to be the most efficient PS removing 58.98% of biofilm biomass (*P* < 0.001). When compared to classical irrigants, Zn(II)e_6_Me was not as effective as NaOCl, the treatment that causes the higher damage, regardless of the type of biofilm (**Figure 3**; right panels). In fact, Zn(II)e_6_Me (with an irradiation period of 90 s) was more effective in removing *E. faecalis* biofilm than EDTA or CHX (**Table 2** and **Figure 3**; upper panel). Zn(II)e_6_Me reveal the same effect of CHX or EDTA treatment toward *C. albicans* biofilms (90 s) and toward mixed biofilms, either with 60 s or with 90 s of irradiation (**Table 2** and **Figure 3**; middle and lower panels).

**Table 2:** Statistical analysis^(1)^ of the efficiency of Zn(II)e_6_Me against microbial biofilms in comparison with classical irrigants in the clearance of *E. faecalis, C. albicans*, and mixed biofilms.

	*E. faecalis*		*C. albicans*		Mix	
	60 s	90 s	60 s	90 s	60 s	90 s
Zn(II)e_6_Me vs. NaOCl	^∗∗∗^	^∗∗∗^	^∗∗∗^	^∗∗^	^∗∗^	^∗∗^
Zn(II)e_6_Me vs. CHX	ns	^∗∗∗^	ns	ns	ns	Ns
Zn(II)e_6_Me vs. EDTA	ns	^∗^	^∗^	ns	ns	ns

*^(1)^Differences were analyzed by Student’s *t*-test using Prism software and considered significant at *P*-values of < 0.05. ^∗^*P* < 0.05; ^∗∗^*P* < 0.01; ^∗∗∗^*P* < 0.001, ns: no significant difference.*

### Biofilms Disturbance by aPDT in the Presence of Zn(II)e_6_Me and NaOCl

The study of biofilms morphology was performed after 48 h of biofilm maturation, because 48 h- and 72 h-biofilms had similar morphologies. The changes observed in the biofilm organization developed for 48 h when treated with Zn(II)e_6_Me and NaOCl (the classical irrigant with the best outcome) were compared with the untreated biofilms (control).

**FIGURE 4:**
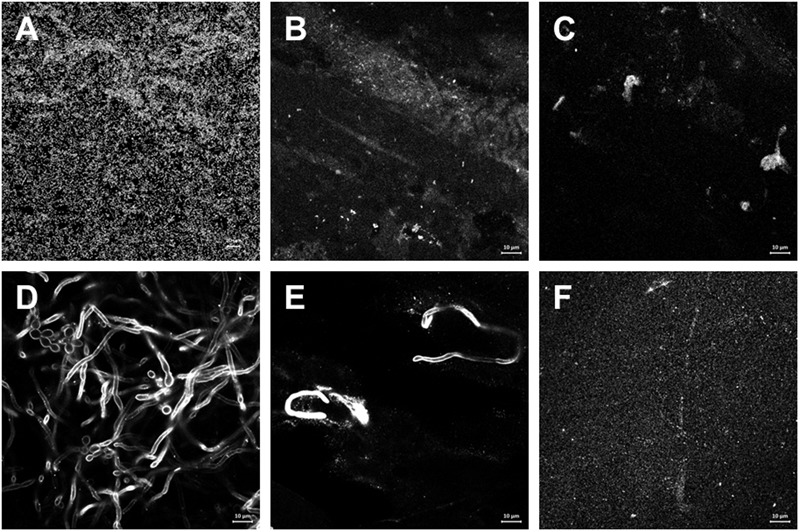
**Effect of aPDT with Zn(II)e_6_Me as photosensitizer compared with the classical irrigant NaOCl in the morphology of monospecies biofilms.** Endodontic *in vitro* 48 h-biofilms of *E. faecalis* and *C. albicans* were obtained and treated as described under Materials and Methods. **(A–C)** *E. faecalis* was stained with Syto 13 Green Fluorescent Nucleic Acid Stain. **(D–F)** *C. albicans* was probed with polyclonal primary antibody and with anti-rabbit secondary antibody Alexafluor^®^594. Representative images of biofilms untreated **(A,D)**, treated with Zn(II)e_6_Me as PS activated for 90 s **(B,E)**, and treated with NaOCl **(C,F)**, were obtained with a Carl Zeiss Cell Observer Spinning Disk with Alpha Plan-Apochromat objective (100×).

Zn(II)e_6_Me eliminated most of the *E. faecalis* (**Figures 4A,B**) but *C. albicans* preparations retained some hyphae and yeast cells (**Figures 4D,E**). Otherwise, NaOCl eliminated all the cells adhered to the glass slide, either in *E. faecalis* or *C. albicans* biofilms (**Figures 4C,F**).

**FIGURE 5:**
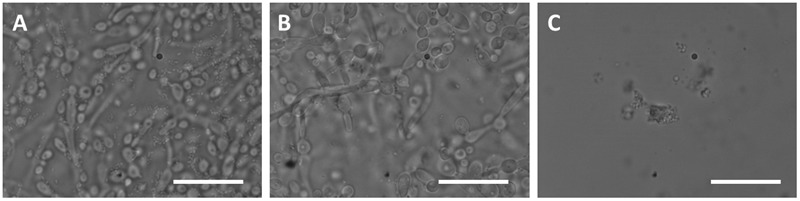
**Effect of PDT with Zn(II)e_6_Me as photosensitizer compared with the classical irrigant NaOCl in the morphology of mixed biofilm.** The images **(A)** untreated controls, **(B)** Zn(II)e_6_Me activated during 90 s, and **(C)** with NaOCl, were obtained with using an Olympus BX-40 microscope at 1000× total magnification. Images were recorded at different time periods on an Olympus C-200 digital camera. Bars: 20 μm.

Using light microscopy, it was observed that while NaOCl lead to an almost complete loss of living cells (**Figure 5C**), aPDT with Zn(II)e_6_Me resulted in a mixed biofilm with less *E. faecalis* cells and less *C. albicans* hypha, with a predominance of pear shaped cells (**Figure 5B**), when compared with the morphology of the untreated mixed biofilm (**Figure 5A**).

### Ultrastructure Modification of Microbial Cells in Biofilms

**FIGURE 6:**
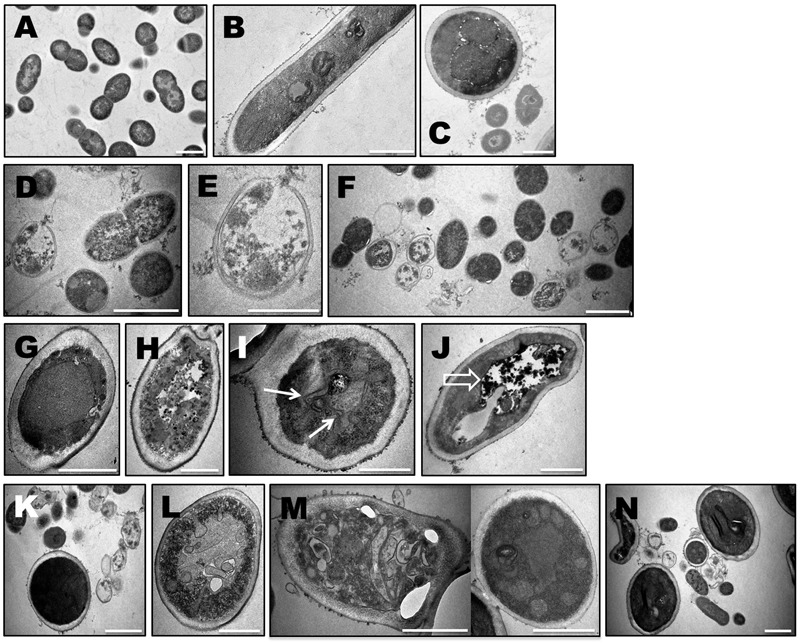
**Ultrastructural modification of microbial cells upon antimicrobial photodynamic therapy (aPDT) with Zn(II)e_6_Me as photosensitizer.** 48 h- biofilms of *E. faecalis, C. albicans* and mixed biofilms with both microrganisms untreated **(A–C)** and treated with aPDT with Zn(II)e_6_Me as photosensitizer (*E. faecalis* biofilm: **D–F**), (*C. albicans* biofilm: **G–J**), (mixed biofilms: **K–N**). Solid arrows indicate peculiar intracellular membrane arrangements probably corresponding to endoplasmic reticulum whorls and open arrows depicted large vacuoles with electrodense materials. Bars, 1000 nm, except for E and I, 500 nm.

The different morphological aspects observed in the fluorescence confocal microscopy lead us to study the ultrastructural changes using TEM. After several attempts, it was realized that the remainings of the biofilms (either monospecies or mixed biofilm) treated with NaOCl were so drastically damaged that no signs of cells were observed in the epoxy resin blocks sections (data not shown). The ultrastructural modification of bacterial and fungal cells were studied in biofilms exposed to Zn(II)e_6_Me with an activation period of 90 s. In *E. faecalis* monospecies biofilm it was observed the existence of cell wall “ghosts”, i.e., bacterial cell wall forming a structure with typical morphology of *E. faecalis*, without its intracellular content (**Figures 6A,D–F**). The complexity of the cellular ultrastructure of *C. albicans*, a eukaryote, allowed the observation of induced modifications. Most of the yeast cells showed an atypical irregular cell wall thickness and the cytoplasmic membrane integrity was lost, with cell membrane invaginations (**Figure 6G**), caused by 90 s of Zn(II)e_6_Me-aPDT treatment. The cell membrane was damaged and the cell wall surface was rougher (**Figure 6H**) than in control cells (**Figure 6B**). Abnormal intracellular membrane arrangements probably corresponding to endoplasmic reticulum (ER) whorls (**Figure 6I**) were also observed. Some *C. albicans* cells exhibited big vacuoles with electrodense materials (**Figure 6J**).

A general view of the mixed biofilms showed *E. faecalis* cell wall structures devoid of the intracellular content and irregular *C. albicans* cell walls (**Figure 6K**). In fact, in mixed biofilms microbial cells ultrastructural modifications were similar to those observed in monospecies biofilms, including invaginations of the cell membrane found in *C. albicans* cells (**Figure 6L**). Additionally, in mixed biofilms, *C. albicans* cells showed persistent extracellular vesicles, at the surface of the cell wall, with different sizes and shapes (**Figure 6M**). In **Figure 6N** it is also possible to observe several ultrastructural features of mixed biofilms treated with Zn(II)e_6_Me by PDT: in the extracellular matrix, besides the spread of electrodense materials typical of a biofilm matrix, fragments of membranes or of fibrous materials were also observed, which were not observed in *E. faecalis–C. albicans* mixed biofilms untreated (**Figure 6C**); also, the cytoplasm of *E. faecalis* showed electrodense agglomerates and some fungal cells showed a twisted irregular shape.

## Discussion

The aim of this work was to compare the efficacy of Zn(II)e_6_Me to disturb *in vitro* models of endodontic biofilms comparatively with three other PSs, TBO, RB, and TMPyP, and also with endodontic classical irrigants. For this, monospecies biofilms of *E. faecalis* and of *C. albicans* were used, together with a mixed biofilm model with both microrganisms. The main conclusion is that Zn(II)e_6_Me had a better antimicrobial efficacy than the clinically used PSs, TBO, and RB. Although the efficacy of Zn(II)e_6_Me and TMPyP is similar, one of the main advantage of using Zn(II)e_6_Me is its availability from natural sources, associated to a lower toxicity in the total absence of light. It also presented the same antimicrobial potential than the clinically used classical irrigants, CHX and EDTA. It is worth mentioning that TBO (Seal et al., 2002; Bergmans et al., 2008; Rios et al., 2011) is available in the market under the name of Fotosan^®^ agent (Gambarini et al., 2011; Rios et al., 2011) and RB has been widely studied (Shrestha et al., 2012, 2014; Persadmehr et al., 2014). As expected, 3% NaOCl had the best final outcomes. In fact, NaOCl at different concentrations, is considered an excellent irrigant solution in endodontics (Jeansonne and White, 1994; Siqueira et al., 2007; Mohammadi, 2008), nevertheless it also displays high toxicity levels toward the host tissues (Estrela et al., 2002; Önçaǧ et al., 2003; Trevino et al., 2011; Wang et al., 2015).

The antimicrobial effect of aPDT is dependent both on the cellular localization of the PS, which may be determined by its physicochemical properties (Castano et al., 2004) and on the diffusion of singlet oxygen that should be sufficient to inactivate microrganisms structures and biomolecules. There have been several reports on the use of aPDT to kill both yeast and bacteria, however, fungi are much more complex targets than bacteria. Nevertheless, similarities with mammalian cells should be considered and this may indicate the use of cationic PSs, rather than their anionic counterparts, since the latter exhibit facile uptake by mammalian cells (Bonnett, 1995). The biochemical and functional effects of photosensitization include peroxidation of lipids, resulting in cell membranes disruption, lysosomes and mitochondria lysis and consequently autophagy (Schuck et al., 2014). The phenothiaziniums, such as TBO and MB, are known to target plasma membrane of yeast and bacteria (de Melo et al., 2013; Baltazar et al., 2015); TBO was described as increasing cell wall permeability (Wainwright et al., 1997), whereas MB produces bacterial DNA damage (Menezes et al., 1990).

The use of cholorophylls in endodontic root canal treatment was previously described (Mohammadi et al., 2013). There are evidencies showing that clorophyll present in green tea can be used in endodontic root canal treatment due to its antibacterial effects (Horiba et al., 1991). In this work, we describe Zn(II)e_6_Me, obtained from the natural chlorophyll a, as a encouraging PS candidate displaying consistent antimicrobial outcomes. The ultrastructural study of microbial cells upon aPDT demonstrated that using Zn(II)e_6_Me as PS, results in the irreversible damage of *E. faecalis* cells (mono and dual-species biofilms), displaying ‘cell ghosts’, empty of its cellular content but with almost intact cell walls. The presence of these inactive “ghost” cells was corroborated by the biomass loss assessed by SR assay. Before, it was described that *E. faecalis* elimination with aPDT resulted in bleb formations suggestive of damage of membrane components (López-Jiménez et al., 2015), shrunken, bacterial cell diameter reduction, rough and fractured appearance of the bacterial cells (Cheng et al., 2012). It was also described the presence of bacterial cell membrane shriveling and alterations including loss of cocci or bacilli shape, grooves on the cell surface and draining of the intracellular components (Garcez et al., 2013). According to our observations, *E. faecalis* cell wall destruction was sporadic and not a massive one, which appear to indicate that the induced damage was directed to proteins and/or lipids of the cytoplasmic membrane, resulting in the leakage of cellular contents, as described by others (Girotti, 2001). It is also known that the extension of biochemical changes induced by aPDT is dependent on the PS nature and on the irradiation period (Dai et al., 2009). In this study the aPDT proceeded during a short period (60 or 90 s), which can justify the punctual cell wall destruction in *E. faecalis*.

In *C. albicans* cells in monospecies biofilms and in *C. albicans* cells in mixed biofilms, it was noticed several changes in the cellular organization, with a cytoplasmic membrane disruption, vacuoles morphology and organelles damage including signals of autophagy (e.g., ER whorls, and organelles inclusion in vacuoles) as described by others (Prates et al., 2011; Schuck et al., 2014). The intracellular damage induced by aPDT with Zn(II)e_6_Me is probably dependent on the entry of this PS, since this feature is crucial for aPDT efficacy and outcomes (Hamblin and Hasan, 2004; Baltazar et al., 2015). This lead us to speculate that the pre-incubation period of biofilms with PSs during 15 min in total absence of light, before the short irradiation period (60 and 90 s) most certainly contributed for the interaction between the PS and the cell. This would lead to intracellular PS distribution (due to its hydrophobic nature), impacting in the genesis of the intracellular damage observed. The questions raised by these observations highlight the importance of future further studies to unravel the intracellular distribution of Zn(II)e_6_Me. The Zn(II)e_6_Me antimicrobial potential, that we showed by quantification of biofilm biomass loss and by a microscopic study of the biofilm morphology and of the cellular ultrastructure, leads to the importance of defining the mechanism by which this modified chlorophyll affects the endodontic biofilms.

Based on this, further research will be mandatory to improve the antimicrobial efficacy of aPDT in the root canal system, such as the ones recently published (Tennert et al., 2015; Cieplik et al., 2016) using human tooth models, ultimately leading to an optimization of light delivery and new PS formulations.

## Author Contributions

PD, IM, JS, FC, and TG were responsible for the conception and design of the study, and for the analysis and interpretation of data; PD, CF, FC, and MM did most of the lab work and analysis of data; PD and TG did most of the manuscript writings; MF, MN, MU, and KdO extracted, modified and analyzed two of the PDT compounds; all the authors contributed equally to the revision of the manuscript and approved the final version to be submitted.

## Conflict of Interest Statement

The authors declare that the research was conducted in the absence of any commercial or financial relationships that could be construed as a potential conflict of interest.
